# Recurrent Vertigo of Childhood Revisited: Clinical Findings Supporting a Developmental Vestibular Migraine Spectrum

**DOI:** 10.3390/audiolres16050149

**Published:** 2026-09-30

**Authors:** Giacinto Asprella Libonati, Rudi Pecci, Giada Cavallaro, Fernanda Asprella Libonati, Paolo Pagnini

**Affiliations:** 1 Otolaryngology Unit, Madonna Delle Grazie Hospital of Matera, 75100 Matera, Italy; giada.cavallaro@asmbasilicata.it; 2 Audiology Clinic, Azienda Ospedaliero Universitaria Careggi, 50134 Florence, Italy; peccirudi@gmail.com (R.P.); paolo.pagnini@libero.it (P.P.); 3 Otolaryngology Unit, Engles Profili Hospital, AST Ancona, 60044 Fabriano, Italy; fernandasprella@gmail.com

**Keywords:** vestibular migraine, recurrent vertigo of childhood, pediatric vertigo, migraine diathesis, benign paroxysmal torticollis

## Abstract

Background: Complaints of vertigo and dizziness are frequently encountered in childhood and may coexist with migraine-associated features. The present study reassessed a historical pediatric cohort within an expanded phenotypic framework. Methods: This retrospective multicenter descriptive study analyzed 146 children aged 3–12 years evaluated for recurrent vestibular symptoms between 1995 and 2022 at two Italian tertiary neuro-otological referral centers. Clinical history and neuro-otological examination represented the common diagnostic foundation, while age-appropriate audiological evaluation was performed in virtually all children and additional vestibular or imaging investigations were clinically selective. Results: Of the 146 children, 144 fulfilled the 2021 Bárány Society criteria for Recurrent Vertigo of Childhood (RVC), whereas two presented with isolated benign paroxysmal torticollis. None fulfilled the criteria for Vestibular Migraine of Childhood or probable Vestibular Migraine of Childhood. Headache was reported in 15.8% of patients, whereas motion sensitivity, sensory hypersensitivity, autonomic symptoms, and additional migraine-associated developmental syndromes were frequently observed. Conclusions: In this selected tertiary cohort, the clustering of migraine-associated features was compatible with a developmental migraine-related vestibular spectrum. These phenotypic associations do not demonstrate longitudinal disease evolution, and prospective longitudinal studies are required.

## 1. Introduction

Complaints of vertigo and dizziness are frequently encountered in childhood and represent a common reason for referral to pediatric otolaryngology and neuro-otology services [1]. Although peripheral vestibular disorders, neurological conditions, and functional disorders must be considered in the differential diagnosis, increasing evidence indicates that migraine-related mechanisms account for a substantial proportion of recurrent episodic vestibular symptoms in children. Migraine is currently recognized as a complex neurobiological disorder involving altered sensory processing and dysfunction of multiple neuronal networks. Accordingly, vestibular migraine should no longer be regarded simply as migraine associated with vertigo, but rather as one phenotypic expression of a broader migraine diathesis. Vestibular symptoms may occur before, during, after, or independently of headache attacks and may represent the predominant clinical manifestation, particularly during childhood [2,3]. During early life, migraine expression differs substantially from the adult phenotype. Before puberty, headache may be absent, poorly characterized, or clinically overshadowed by episodic non-cephalic manifestations, including recurrent vertigo, benign paroxysmal torticollis, cyclic vomiting, abdominal migraine, motion sickness, and sensory hypersensitivity [4]. Epidemiological and clinical studies have demonstrated strong associations between recurrent vestibular symptoms in children and migraine-related features, such as family history of migraine, motion intolerance, and sensory hypersensitivity, supporting the concept that vestibular manifestations may represent integral components of the migraine spectrum rather than isolated vestibular disorders [5]. To improve diagnostic standardization, the Bárány Society and the International Headache Society introduced in 2021 consensus criteria distinguishing Vestibular Migraine of Childhood (VMC), probable Vestibular Migraine of Childhood (probable VMC), and Recurrent Vertigo of Childhood (RVC) [6] (Table 1). Although this classification represents an important advance, its reliance on headache-related criteria may reduce diagnostic sensitivity in prepubertal children, in whom migraine may manifest predominantly through vestibular and autonomic symptoms [7]. A developmental model of pediatric migraine suggests that different age-related phenotypes, including benign paroxysmal torticollis, cyclic vomiting, abdominal migraine equivalents, recurrent vestibular symptoms, and later vestibular migraine, may represent distinct expressions of an underlying migraine susceptibility. Within this framework, childhood vestibular disorders may reflect an evolving clinical spectrum rather than separate diagnostic entities.

The distinction between vertigo and dizziness is clinically relevant, as pediatric patients frequently lack the vocabulary required to accurately characterize vestibular sensations [2]. The present study reports a 27-year multicenter experience involving 146 children evaluated for recurrent vestibular symptoms at two Italian tertiary referral centers. The aim was to reassess this historical pediatric cohort within an expanded phenotypic framework integrating vestibular manifestations, migraine-associated developmental syndromes, family history, autonomic features, motion sensitivity, and sensory hypersensitivity. We specifically investigated whether children classified predominantly as RVC nevertheless exhibited a clustering of migraine-related features that could support the hypothesis of an early developmental migraine-associated vestibular phenotype.

## 2. Materials and Methods

This retrospective observational study was conducted over a 27-year period (1995–2022) in two tertiary vestibology centers: the SOD Audiologia e Otorinolaringoiatria, Azienda Ospedaliera Universitaria Careggi, Florence and the UOC Otorinolaringoiatria, “Madonna delle Grazie” Hospital, Matera, and UO Otorinolaringoiatria e Vestibologia, Ospedale “Giovanni Paolo II”, ASM Matera, Policoro. The study population consisted of 146 children aged 3–12 years evaluated for recurrent vestibular symptoms with recurrent vestibular symptoms (Table 2). Patients were included if they presented with recurrent spontaneous vestibular symptoms and had available clinical information regarding vestibular manifestations and migraine-related features. Patients with identifiable alternative causes of vertigo, including structural neurological disorders, congenital or acquired vestibular diseases, significant hearing impairment, inflammatory or infectious conditions, or incomplete clinical documentation were excluded. Categorical variables were summarized as absolute frequencies and percentages. Continuous variables were described using ranges, and where available, means or medians. No inferential statistical comparisons were performed because the study was designed as a retrospective descriptive cohort analysis. Because the study was designed as a retrospective descriptive phenotypic analysis rather than an analytical study of causal associations, no multivariable adjustment for potential confounding variables was performed; observed associations should therefore be interpreted as phenotypic associations rather than causal relationships. The cohort included 95 patients from Florence and 51 from Matera, with a female predominance. At presentation, 144 children exhibited recurrent vestibular symptoms, classified as spinning vertigo or non-spinning dizziness. Four of these patients also presented with benign paroxysmal torticollis. Two additional children presented with isolated recurrent benign paroxysmal torticollis, interpreted according to Pagnini’s model as a migraine-related manifestation of the descending vestibulo-cervical pathway [8]. Headache was reported in 23 patients (15.8%) and was generally mild to moderate in intensity. All patients were initially classified according to the 2021 Bárány Society and International Headache Society consensus criteria for pediatric vestibular disorders, including Vestibular Migraine of Childhood (VMC), probable Vestibular Migraine of Childhood, and Recurrent Vertigo of Childhood (RVC). Among the 146 patients, 144 fulfilled the criteria for RVC, whereas the two children with isolated recurrent torticollis did not meet the formal Bárány Society criteria for RVC but were included within the expanded migraine-related vestibular spectrum. None met the diagnostic criteria for VMC or probable VMC. Given the potential limitations of the current diagnostic frameworks in pediatric populations, a secondary structured reassessment was performed. Family history of migraine was assessed as a phenotypic variable and was not used as an inclusion criterion. This evaluation included migraine family history, sex, age at symptom onset, vestibular phenotype (spinning vertigo versus non-spinning dizziness), headache characteristics, and the presence of vestibular and extra-vestibular migraine-related precursors. Benign paroxysmal torticollis was considered a vestibular precursor, while cyclic abdominal pain and cyclic vomiting were considered extra-vestibular precursors. Additional probable migraine-related manifestations, including recurrent limb pain, recurrent fever, and infant colic, were also assessed. Features associated with migraine susceptibility, including motion sickness, sensory hypersensitivity, photophobia, phonophobia, osmophobia, dysosmia, and intolerance to motion stimuli, were systematically evaluated. The upper age limit of 12 years was used as an operational criterion reflecting the clinical focus of the historical cohort and an intended pre-/early-pubertal developmental framework; chronological age was not considered equivalent to documented biological pubertal status because Tanner staging or standardized endocrinological assessment was not systematically available.

Because the cohort spans a 27-year period (1995–2022), a uniform instrumental vestibular protocol could not be applied throughout the entire study period. Detailed clinical history and neuro-otological examination represented the diagnostic cornerstone, while instrumental testing primarily contributed to differential diagnosis rather than defining RVC, consistent with the clinical nature of the Bárány Society/IHS framework [6]. Age-appropriate audiological evaluation was performed in virtually all children to exclude significant hearing impairment or cochlear disease. Additional vestibular investigations, including positional testing, videonystagmographic/electronystagmographic assessment, caloric or rotatory testing, and in later years, vHIT and vestibular-evoked myogenic potentials, were performed according to clinical indication, patient age and cooperation, and availability of the technique. Neuroimaging was not systematically performed in all patients; brain MRI and/or targeted imaging was requested when clinical history, neurological or neuro-otological examination, auditory findings, persistence or atypical characteristics of symptoms raised suspicion of a structural neurological or otological disorder. Developmental, behavioral, and learning conditions were not systematically recorded as predefined variables throughout the study period and could therefore not be reliably analyzed retrospectively.

## 3. Results

Among the 146 children, 144 met the Bárány Society criteria for RVC. The remaining two presented with isolated recurrent torticollis, considered a migraine-related vestibulo-cervical phenotype according to Pagnini’s model [9]. None fulfilled the diagnostic criteria for Vestibular Migraine of Childhood or probable Vestibular Migraine of Childhood, despite presenting with a clinical picture strongly suggestive of migraine-related disease. A positive family history of migraine was documented in all patients. The predominant clinical presentation was recurrent vestibular symptoms, observed in almost all patients, while a small subgroup presented with associated vestibulo-cervical manifestations. Specifically, four children experienced both recurrent vestibular symptoms and benign paroxysmal torticollis, and two presented with isolated recurrent torticollis. Overall, six children exhibited a torticollis phenotype. In four patients, torticollis coexisted with recurrent vertigo or dizziness, whereas in two, it represented the isolated clinical expression of a migraine-related vestibulo-cervical disorder. Symptom onset occurred predominantly between the ages of three and eight years. In patients with torticollis, episodes were retrospectively reported to have begun earlier, typically between two and four years of age. A clear female predominance was observed across the cohort, in keeping with known epidemiological patterns of migraine disorders. Headache was present in a minority of cases and was generally mild or moderate in intensity. In most patients, vestibular symptoms preceded the onset of headache, supporting the hypothesis that vestibular manifestations may represent an early expression of migraine biology in childhood. Vestibular episodes were characterized by abrupt onset during normal activity, variable duration ranging from minutes to several hours, and complete spontaneous resolution. Clinical manifestations included motor arrest, imbalance, fear behavior, and in older children, clearly described spinning vertigo. Autonomic symptoms such as pallor, nausea, sweating, and anorexia were frequently observed, whereas vomiting occurred less consistently. In selected cases, parental smartphone recordings documented transient nystagmoid eye movements with peripheral characteristics. Patients with torticollis exhibited episodic tonic rotation and lateral flexion of the neck, with spontaneous resolution and no residual deficits. These episodes often preceded or coexisted with vestibular symptoms, suggesting a shared underlying mechanism. A substantial proportion of patients also presented with extra-vestibular migraine precursors, including recurrent abdominal pain, cyclic vomiting, recurrent limb pain, episodic unexplained fever, and a history of infant colic. Among these, abdominal pain and cyclic vomiting were the most consistently reported and frequently led to repeated negative gastrointestinal evaluations. Markers of migraine susceptibility were highly prevalent. Motion sickness was the most frequent feature, followed by sensory hypersensitivity manifestations such as phonophobia, hyperacusis, osmophobia, dysosmia, photophobia, and intolerance to motion or acceleration stimuli. These traits often preceded vestibular symptom onset and were considered strong indicators of an underlying migraine predisposition.

## 4. Discussion

The coexistence of migraine-associated features in children with RVC should not be interpreted as proof that RVC is an early stage of vestibular migraine. Rather, their clustering within this selected cohort represents a phenotypic association compatible with, and supportive of, a developmental-spectrum hypothesis requiring prospective longitudinal validation. The present 27-year multicenter cohort study provides evidence that a substantial proportion of children classified under the current Bárány Society framework as having Recurrent Vertigo of Childhood (RVC) exhibit clinical features that are compatible with a broader migraine-related vestibular spectrum. Although the 2021 Bárány Society and International Headache Society consensus criteria constitute an important step toward standardized classification of pediatric vestibular disorders, our findings raise the possibility that their current operational structure may not fully capture migraine-related vestibular phenotypes in prepubertal populations, particularly when headache is absent. A key implication of our findings is that migraine-related vestibular disorders in childhood may be better understood within a developmental framework rather than solely as discrete diagnostic entities (Figure 1). The clinical expression of migraine susceptibility may undergo age-dependent transformation, potentially reflecting the maturation of sensory networks, nociceptive pathways, and neuroendocrine regulation [10]. In the prepubertal period, vestibular manifestations may predominate, while headache may be absent, incompletely expressed, or clinically overshadowed by episodic vestibular and autonomic symptoms [11]. This potential dissociation between vestibular symptoms and headache may help explain the low proportion of children fulfilling the current diagnostic criteria for Vestibular Migraine of Childhood despite exhibiting multiple markers of migraine susceptibility [12,13,14,15]. Within this framework, recurrent vertigo of childhood may be compatible with an early developmental phenotype within a broader migraine-related spectrum, although the present study cannot establish that it represents an early stage of vestibular migraine. Similarly, benign paroxysmal torticollis, cyclic vomiting, abdominal migraine equivalents, and later vestibular migraine may represent age-dependent phenotypic expressions associated with migraine susceptibility, rather than necessarily constituting a demonstrated sequential disease trajectory. The transition from a predominantly vestibular phenotype during childhood toward a more multisensory and headache-associated phenotype after puberty may therefore reflect phenotypic maturation, although this hypothesis requires longitudinal confirmation. Puberty may represent an important biological transition in this continuum. Hormonal maturation, particularly the influence of fluctuating gonadal hormones on trigeminovascular activation, sensory processing, and neurotransmitter systems, may modify the clinical expression of migraine susceptibility [16]. Although female predominance is generally considered more evident after puberty, females were also overrepresented in our prepubertal referral cohort. This finding may reflect referral patterns, selection factors, or an earlier sex-related modulation and should be interpreted cautiously [17,18,19]. The vestibular phenotype itself appears heterogeneous, suggesting variable involvement of different components of the central vestibular network. Some children experience predominantly rotatory vertigo, characterized by a clear perception of spinning, whereas others report imbalance, rocking sensations, motion intolerance, or nonspecific dizziness without true spinning vertigo. These differences may reflect different clinical expressions of altered multisensory vestibular integration [20,21]. They should not necessarily be interpreted as evidence for distinct disorders, but rather as phenotypic variation that may occur within a migraine-related susceptibility framework affecting the vestibulo-thalamo-cortical, cerebellar, and brainstem networks. This concept may also explain why the terminology of “vertigo” alone fails to capture the full spectrum of pediatric migraine-related vestibular symptoms. The distinction between vertigo and dizziness is therefore clinically relevant, as pediatric patients frequently lack the vocabulary required to accurately characterize vestibular sensations [9]. A broader assessment incorporating the quality of vestibular symptoms, motion sensitivity, sensory hypersensitivity, autonomic activation, and developmental migraine precursors may improve diagnostic sensitivity and help identify children with a migraine-related vestibular phenotype. Overall, our findings support the hypothesis that pediatric migraine-related vestibular disorders may constitute a dynamic developmental spectrum characterized by age-dependent phenotypic variation rather than static disorders defined by the simultaneous presence of vestibular symptoms and headache [22,23,24]. Such a framework may provide a biologically plausible model for the clinical heterogeneity observed in childhood, while its implications for longitudinal evolution across developmental stages remain to be established. In our cohort, headache at presentation was reported in only 15.8% of children, despite the presence of a consistent migraine-associated phenotype characterized by positive family history of migraine, motion sickness, sensory hypersensitivity, autonomic activation during attacks, and multiple developmental episodic syndromes historically recognized as migraine precursors. This dissociation between headache and vestibular symptomatology is compatible with the possibility that during early life, migraine susceptibility may preferentially manifest through vestibular and autonomic pathways rather than through the classic headache phenotype. The high prevalence of motion sickness and sensory hypersensitivity observed in our patients further supports their potential role as markers of altered central sensory processing. Moreover, the frequent coexistence of vestibular symptoms with benign paroxysmal torticollis is compatible with the possibility that vestibular and cervico-motor paroxysmal disorders may share features of a broader migraine-related developmental phenotype affecting vestibulo-ocular and vestibulo-spinal pathways. In this context, benign paroxysmal torticollis, cyclic vomiting, abdominal migraine equivalents, and recurrent vertigo may be considered within a common migraine-spectrum framework, although the present study cannot establish that they represent sequential or mechanistically identical manifestations. The absence of any patient fulfilling the criteria for Vestibular Migraine of Childhood or probable Vestibular Migraine, despite the presence of multiple migraine-associated features, may indicate that the current Bárány Society criteria do not capture all possible migraine-related vestibular phenotypes in early life, particularly those in which headache is absent. Our developmental model expands the original classification proposed by Pagnini et al. [8] (Figure 2) by considering the possibility that migraine-related vestibular disorders may exhibit age-dependent phenotypic variation rather than representing necessarily distinct nosological entities. From a clinical perspective, these findings support the consideration of migraine susceptibility markers, including family history, developmental episodic syndromes, sensory hypersensitivity, motion intolerance, and autonomic symptomatology, in the diagnostic evaluation of children presenting with recurrent vertigo. This approach may facilitate the recognition of a migraine-related vestibular phenotype and may help guide clinical assessment, while its prognostic implications for later migraine phenotypes remain to be determined. This study has several limitations. Its retrospective design and the 27-year observation period resulted in heterogeneous clinical documentation and instrumental assessment; a uniform modern vestibular test battery was not performed in every patient, and neuroimaging was clinically selective rather than systematic. Because the cohort was derived from tertiary neuro-otological referral centers, referral enrichment and selection bias cannot be excluded; accordingly, the prevalence of individual clinical features, including the 100% prevalence of positive migraine family history, should not be extrapolated directly to the general pediatric population. The retrospective descriptive design precluded formal adjustment for potential confounding factors, and developmental, behavioral, and learning conditions were not systematically recorded. Finally, the developmental interpretation is based on cross-sectional phenotypic associations rather than prospective longitudinal follow-up, and therefore cannot demonstrate individual transitions from RVC to vestibular migraine or other migraine-related disorders. Pediatric recurrent vestibular disorders may represent heterogeneous clinical expressions associated with migraine susceptibility. In this selected 27-year tertiary cohort, the clustering of vestibular symptoms, autonomic features, developmental episodic syndromes, family history, motion sensitivity, and sensory hypersensitivity was compatible with a developmental migraine-related vestibular spectrum. These findings should be interpreted as phenotypic associations rather than causal or longitudinal evidence. Prospective longitudinal studies are required to determine whether these childhood phenotypes predict the subsequent development of vestibular migraine or other migraine-related disorders.

## Figures and Tables

**Figure 1:**
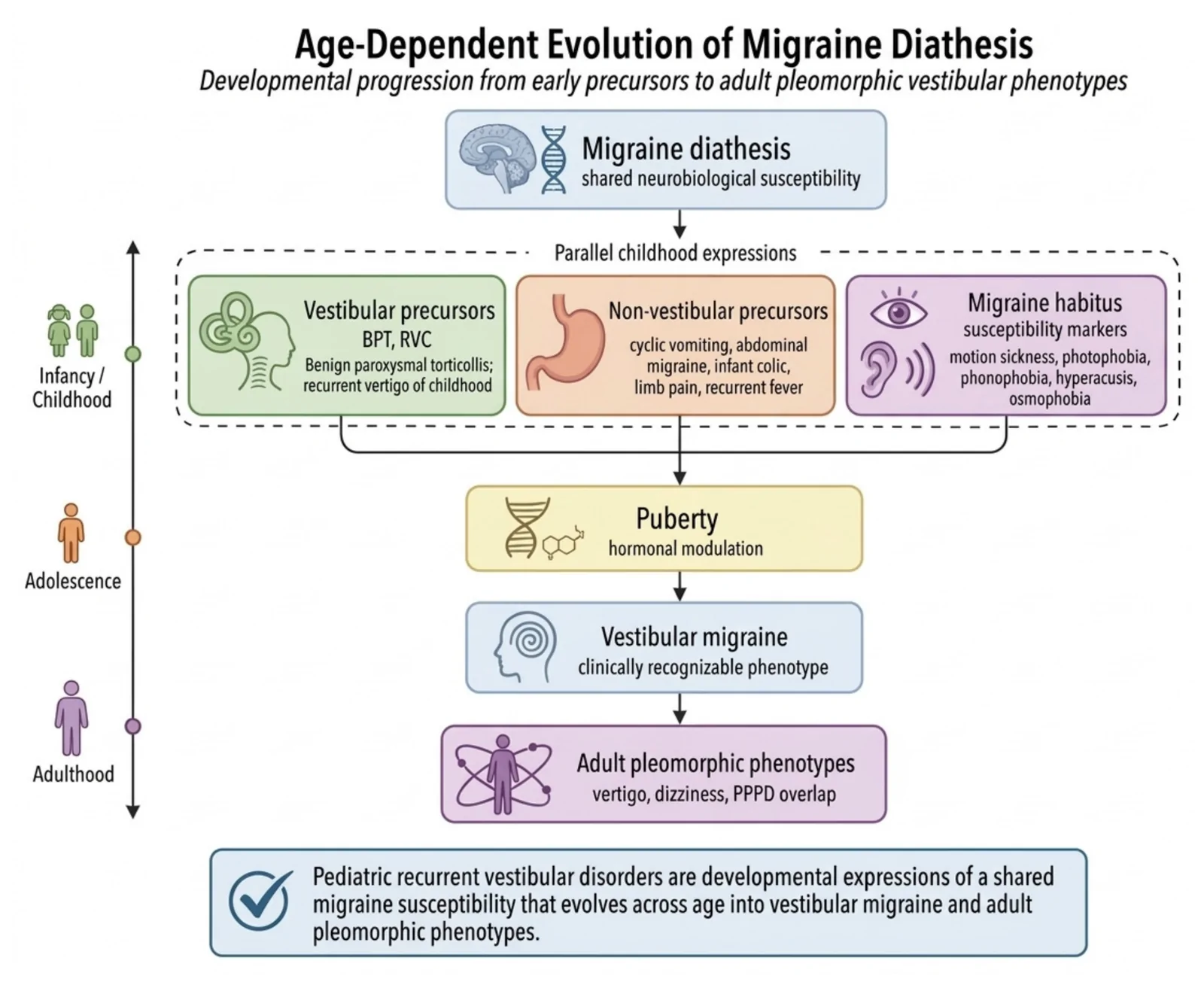
Age-dependent evolution of migraine diathesis. Vestibular precursors, non-vestibular precursors, and migraine habitus are represented as parallel and potentially coexisting childhood expressions and susceptibility traits, rather than obligatory sequential stages.

**Figure 2:**
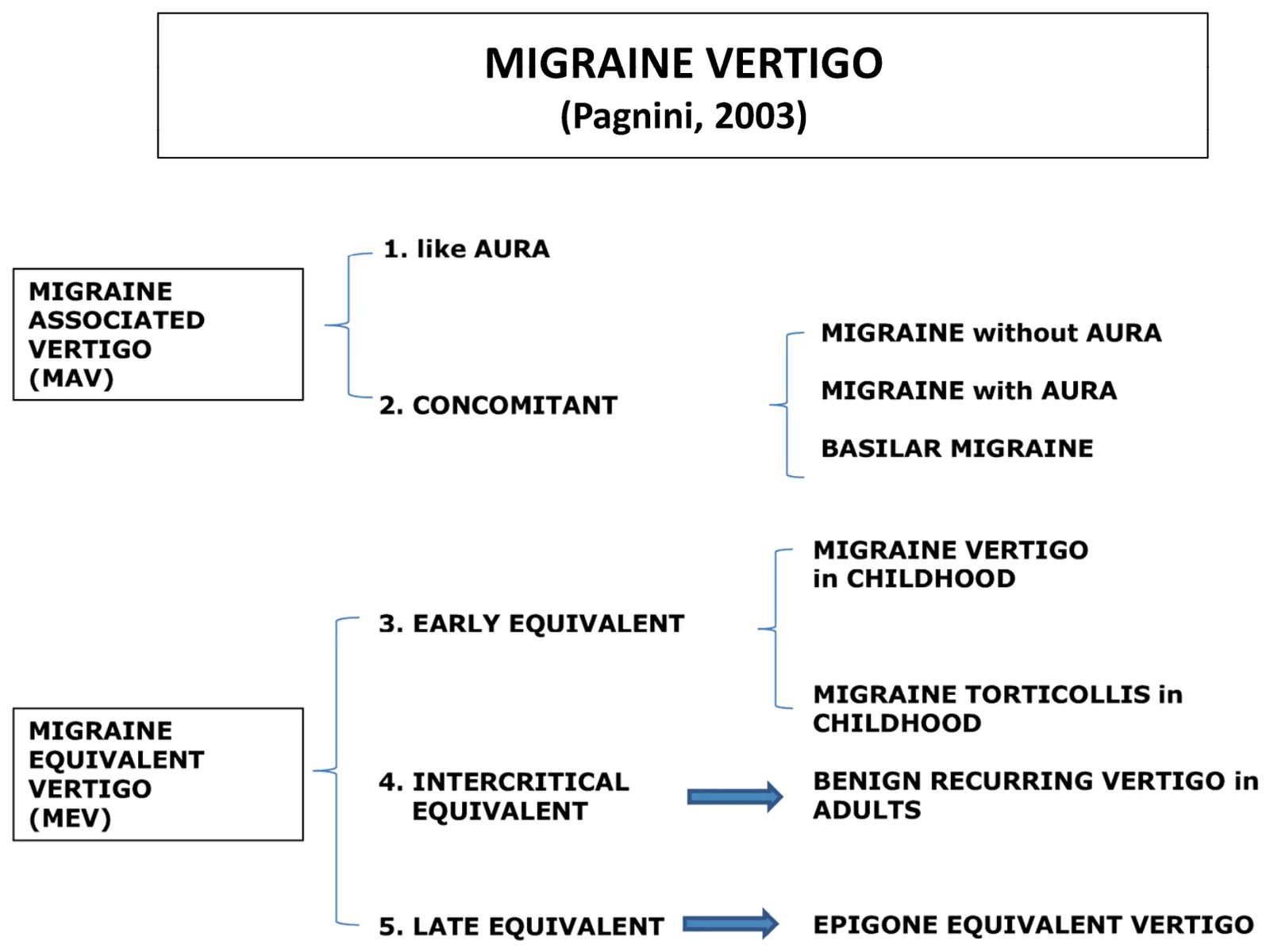
Original classification of migraine-related vertigo proposed by Pagnini et al. [8]. Reproduced from Pagnini et al. [8] with permission from the authors.

**Table 1:** Diagnostic criteria for Vestibular Migraine of Childhood, probable Vestibular Migraine of Childhood, and Recurrent Vertigo of Childhood according to the 2021 Bárány Society–International Headache Society consensus.

*Vestibular Migraine of Childhood (VMC)*
At least five episodes with vestibular symptoms of moderate or severe intensity, lasting between five minutes and 72 h. A current or past history of migraine with or without aura. At least half of episodes are associated with at least one of the following three migraine features: Headache with at least two of the following four characteristics: (a) One-sided location; (b) Pulsating quality; (c) Moderate or severe pain intensity; (d) Aggravation by routine physical activity. Photophobia and phonophobia. Visual aura. Age < 18 years. Not better accounted for by another headache disorder, vestibular disorder, or other condition.
*Probable Vestibular Migraine of Childhood (Probable VMC)* At least three episodes with vestibular symptoms of moderate or severe intensity, lasting between five minutes and 72 h. Only one of the criteria B and C for Vestibular Migraine of Childhood. Age < 18 years. Not better accounted for by another headache disorder, vestibular disorder, or other condition.
*Recurrent Vertigo of Childhood (RVC)* At least three episodes with vestibular symptoms of moderate or severe intensity, lasting between 1 min and 72 h. None of the criteria B and C for Vestibular Migraine of Childhood. Age < 18 years. Not better accounted for by another headache disorder, vestibular disorder, or other condition.

**Table 2:** Demographic characteristics, clinical presentation, migraine-related precursors, and susceptibility markers in the pediatric cohort.

Number of patients	146 (95 Florence + 51 Matera)
Age	3–12 years
Sex	92 F (63.0%)–54 M (37.0%)
Sexual maturity	Not systematically assessed; chronological age ≤12 years was an operational inclusion criterion
Familiarity for migraine	146/146 (100%); interpret cautiously because of referral and selection enrichment in this tertiary cohort
SYMPTOMS	
Vertigo described as a spinning sensation	58/144 (40.3%)
Vertigo (spinning sensation) associated with autonomic symptoms	47/58 (81.0%)
Dizziness	86/144 (59.7%)
associated with autonomic symptoms	53/86 (61.6%)
Headache	23 (15.8%), generally mild to moderate and predominantly reported in children older than 10 years.
Benign paroxysmal torticollis (BPT)	6/146 (4.1%); isolated BPT: 2/146 (1.4%)
Definite extra-vestibular migraine precursors	Cyclic abdominal pain 24 (16.4%) Cyclic vomiting 18 (12.3%)
Probable migraine-related precursors	Recurrent limb pain 16 (11.0%) Recurrent fever 10 (6.8%) Infant colic 32 (21.9%)
Migraine-associated susceptibility features	Motion sickness and/or intolerance to acceleration 104 (71.2%) Phonophobia, hyperacusis 63 (43.2%) Osmophobia, dysosmia 39 (26.7%) Photophobia 55 (37.7%)

## Data Availability

The original contributions presented in this study are included in the article. Further inquiries can be directed to the corresponding author.
